# Immediate Prosthetic Breast Reconstruction after Nipple-Sparing Mastectomy: Traditional Subpectoral Technique versus Direct-to-Implant Prepectoral Reconstruction without Acellular Dermal Matrix

**DOI:** 10.3390/jpm11020153

**Published:** 2021-02-22

**Authors:** Gianluca Franceschini, Lorenzo Scardina, Alba Di Leone, Daniela Andreina Terribile, Alejandro Martin Sanchez, Stefano Magno, Sabatino D’Archi, Antonio Franco, Elena Jane Mason, Beatrice Carnassale, Federica Murando, Armando Orlandi, Liliana Barone Adesi, Giuseppe Visconti, Marzia Salgarello, Riccardo Masetti

**Affiliations:** 1 Department of Woman and Child Health and Public Health, Division of Breast Surgery, Fondazione Policlinico Universitario Agostino Gemelli IRCCS, Università Cattolica del Sacro Cuore, Largo Agostino Gemelli, 8, 00168 Rome, Italy; lorenzoscardina@libero.it (L.S.); alba.dileone@policlinicogemelli.it (A.D.L.); daniela.terribile@policlinicogemelli.it (D.A.T.); martin.sanchez@hotmail.it (A.M.S.); stefano.magno@policlinicogemelli.it (S.M.); sabatinodarchi@gmail.com (S.D.); antonio.franco89@icloud.com (A.F.); elenajanemason@gmail.com (E.J.M.); carnassale.beatrice@gmail.com (B.C.); murandofederica@gmail.com (F.M.); riccardo.masetti@policlinicogemelli.it (R.M.); 2 Comprehensive Cancer Center, Multidisciplinary Breast Unit, Fondazione Policlinico Universitario Agostino Gemelli IRCCS, Università Cattolica del Sacro Cuore, Largo Agostino Gemelli, 8, 00168 Rome, Italy; armando.orlandi@policlinicogemelli.it; 3 Department of Woman and Child Health and Public Health, Division of Plastic Surgery, Fondazione Policlinico Universitario Agostino Gemelli IRCCS, Università Cattolica del Sacro Cuore, Largo Agostino Gemelli, 8, 00168 Rome, Italy; liliana.baroneadesi@policlinicogemelli.it (L.B.A.); joevisconti@hotmail.com (G.V.); marzia.salgarello@policlinicogemelli.it (M.S.)

**Keywords:** breast cancer, nipple-sparing mastectomy, immediate breast reconstruction, acellular dermal matrix (ADM), aesthetic and oncological outcomes, quality of life

## Abstract

Background: The aim of this study was to compare outcomes of immediate prosthetic breast reconstruction (IPBR) using traditional submuscular (SM) positioning of implants versus prepectoral (PP) positioning of micropolyurethane-foam-coated implants (microthane) without further coverage. Methods: We retrospectively reviewed the medical records of breast cancer patients treated by nipple-sparing mastectomy (NSM) and IPBR in our institution during the two-year period from January 2018 to December 2019. Patients were divided into two groups based on the plane of implant placement: SM versus PP. Results: 177 patients who received IPBR after NSM were included in the study; implants were positioned in a SM plane in 95 patients and in a PP plane in 82 patients. The two cohorts were similar for mean age (44 years and 47 years in the SM and PP groups, respectively) and follow-up (20 months and 16 months, respectively). The mean operative time was 70 min shorter in the PP group. No significant differences were observed in length of hospital stay or overall major complication rates. Statistically significant advantages were observed in the PP group in terms of aesthetic results, chronic pain, shoulder dysfunction, and skin sensibility (*p* < 0.05), as well as a trend of better outcomes for sports activity and sexual/relationship life. Cost analysis revealed that PP-IPBR was also economically advantageous over SM-IPBR. Conclusions: Our preliminary experience seems to confirm that PP positioning of a polyurethane-coated implant is a safe, reliable and effective method to perform IPBR after NSM.

## 1. Introduction

Immediate prosthetic breast reconstruction (IPBR) is considered as an integral part of the surgical treatment of patients undergoing nipple-sparing mastectomy (NSM) for breast cancer, as it positively affects psychological health, sexuality, body image, and self-esteem.

Traditionally, IPBR has been performed by placement of the prosthetic implant in a submuscular (SM) pocket created beneath the pectoralis major muscle, in order to protect the integrity of the implant and reduce its visibility and palpability [1,2]. Although this technique has shown increasingly good results, it still yields a higher risk of undesirable outcomes such as significant postoperative pain, injury-induced muscular deficit, breast animation deformity, lateral deviation of the breast mound with poor inframammary fold definition, and insufficient lower pole fullness [3,4].

In recent years, placement of the implant in a prepectoral (PP) plane has been increasingly employed. When this technique is performed, the implant is usually covered with an acellular dermal matrix (ADM) to shield it in the subcutaneous space underneath the skin flaps; however, the use of ADM has been reported to increase risks of seroma, infection, and skin/nipple-areola complex (NAC) necrosis, and associated with higher medical costs [1]. To limit these inconveniences, the use of implants with a special micropolyurethane-foam-coated shell surface (microthane) that does not require ADM coverage has recently been proposed [2,5].

The aim of this study was to compare outcomes between traditional SM-IPBR and a PP technique using microthane implants without ADMs in patients undergoing NSM.

## 2. Materials and Methods

After approval from the Institutional Review Board of our hospital, a retrospective review of the medical records of breast cancer patients who underwent NSM followed by IPBR over the two-year period of January 2018–December 2019 was performed. Patients treated before January 2018 were not enrolled because before that date, PP-IPBR in our institution was routinely performed with ADMs, which would have added heterogeneity to our population.

Patients were divided into two cohorts based on the site of implant placement: in SM-IPBR, anatomical textured implants were positioned in the subpectoral pocket according to a previously described standardized technique, while in PP-IPBR, a definitive Polytech implant with a micropolyurethane-foam-coated shell surface was placed in the subcutaneous plane [5,6].

### 2.1. Operative Protocol and Surgical Technique

A complete preoperative workup including clinical assessment, ultrasonography, mammography, breast MRI, and disease staging was performed in all patients; surgical planning was always discussed in a multidisciplinary dedicated surgery board. Common indications to NSM included large tumor-to-breast size, inability to obtain clear surgical margins with breast-conserving surgery, extensive or multicentric disease, contraindications to adjuvant radiotherapy, and patient preference; absolute contraindications to NSM with both types of reconstruction were inflammatory carcinoma, a locally advanced tumor infiltrating the skin or NAC, and previous radiotherapy. Obesity (BMI > 30 kg/m^2^), large breasts with severe ptosis, and active smoking were considered as relative contraindications due to the increased risk of skin or NAC necrosis, breast asymmetry, and nipple displacement [2,3,4,5,6]. Bilateral NSM was performed in patients with a bilateral breast tumor or in women with unilateral disease and a high risk of contralateral breast cancer, such as BRCA mutation carriers.

A specific algorithm shared with the plastic surgeons, based on anamnestic, morphological, functional, and oncological criteria, was used to define the most appropriate reconstruction technique [7,8]. The Rancati classification, based on digital mammographic imaging, was used to predict thickness of post-mastectomy skin flaps [9].

In the vast majority of cases, NSM was carried out through a radial incision on the external quadrants; axillary or inframammary crease incisions were used only in selected cases. Skin flaps and NAC were progressively elevated from glandular tissue. The entire gland was then separated from the muscular plane and removed, preserving the superficial pectoralis fascia. An accurate circumferential palpation of the surgical cavity after removal of the gland was always performed to rule out the possibility of residual breast tissue. Intraoperative pathology evaluation of retroareolar tissue was performed in all cases to confirm secure margins. The removed gland was always weighed to better determine the subsequent reconstruction volume.

**Figure 1:**
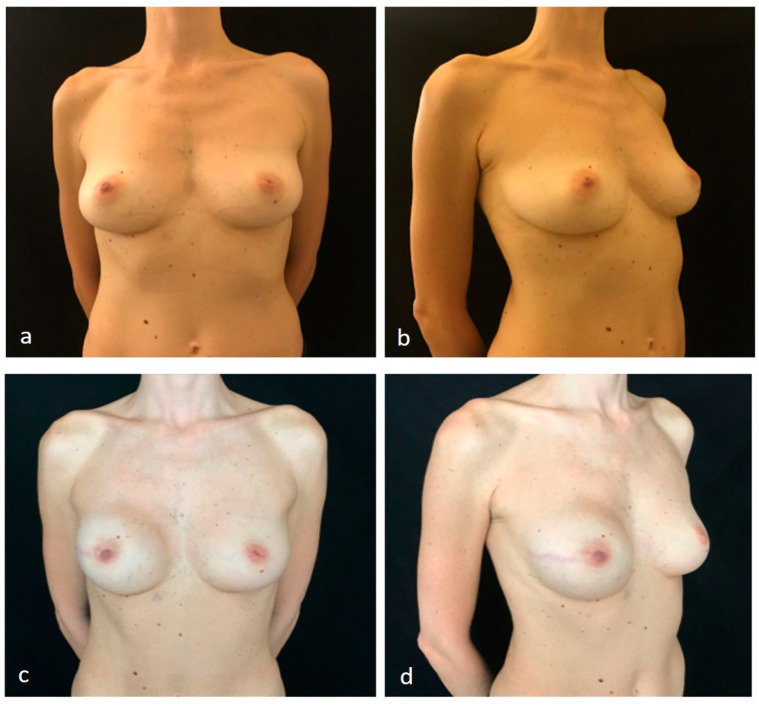
A case of nipple-sparing mastectomy and direct-to-implant prepectoral reconstruction without acellular dermal matrix. (**a**,**b**) Preoperative pictures of a 43-year-old right-breast cancer patient for whom right nipple-sparing mastectomy and direct-to-implant prepectoral reconstruction without acellular dermal matrix were planned. (**c**,**d**) Six-month postoperative pictures after right nipple-sparing mastectomy through a radial lateral incision (mastectomy specimen 190 g) and prepectoral reconstruction using a definitive anatomical implant (Polytech 30746, 295cc) with a micropolyurethane-foam-coated shell surface, placed in the subcutaneous plane.

**Figure 2:**
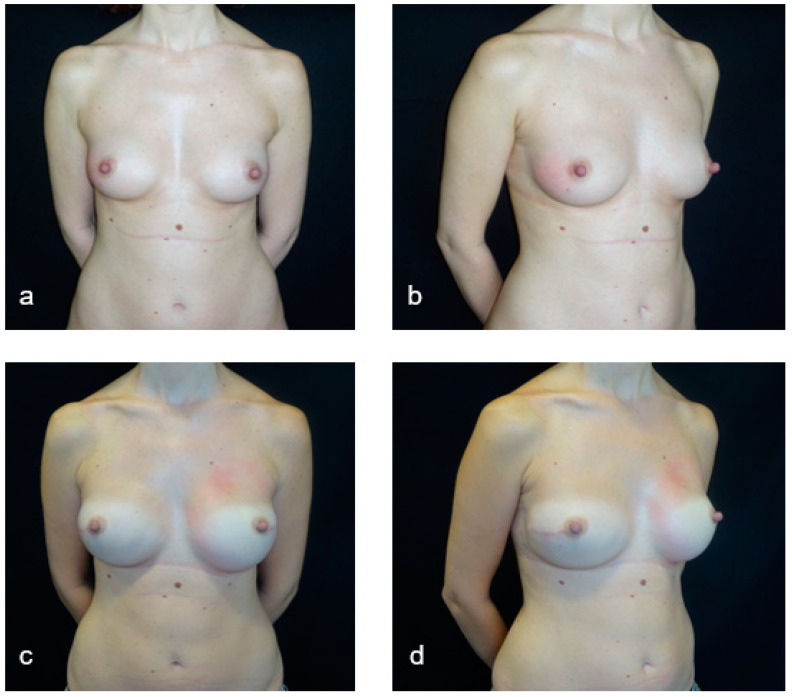
A case of nipple-sparing mastectomy and direct-to-implant submuscular reconstruction. (**a**,**b**) Preoperative pictures of a 47-year-old bilateral-breast cancer patient for whom bilateral nipple-sparing mastectomy and direct-to-implant submuscular reconstruction without acellular dermal matrix were planned. (**c**,**d**) Six-month postoperative pictures after bilateral nipple-sparing mastectomy through a radial lateral incision.

The final decision on the type of reconstructive technique (SM versus PP) was made in the operating room based on flap thickness and perfusion assessment [2,10]. Skin-flap thickness was measured using pliers, and perfusion was assessed using indocyanine green dye fluoroangiography and a photodynamic eye (PDE) imaging system (Figure 1 and Figure 2).

A single-stage SM reconstruction was performed using total coverage of the implant beneath the pectoralis major and serratus anterior [7]; PP-IPBR was realized with the placement of the prosthesis into the same anatomical space of the excised mammary gland [2,5]; textured implants were used for SM-IPBR and Polytech implants with a micropolyurethane-foam-coated shell surface for PP-IBPR [2,5]; and a contralateral procedure to achieve better symmetry was performed when deemed necessary [10,11].

We chose to position a prepectoral implant every time we had good soft-tissue coverage after mastectomy (defined as flap thickness of at least 1 cm and good perfusion with indocyanine green dye fluoroangiography and the photodynamic eye imaging system). In SM-IBPR, we performed a submuscular–subfascial pocket dissection, which allows, with time, a good ptosis. In these cases, any exceeding skin can usually be nicely managed by intraoperative redraping with taping. In SM-IBPR, reduction–augmentation procedures were performed as previously reported.

Two Jackson Pratt drains were always placed in the reconstructive space, usually left in place at the time of hospital discharge and later removed when the amount of fluid collected over 24 h was <30 mL. Patients received levofloxacin at a dosage of 500 mg every 12 h until drain removal and were advised to continue wearing a sports bra for 1 month.

The operative time (from incision to the end of skin suture) and length of hospitalization were recorded.

### 2.2. Clinical Assessment and Statistical Analysis

Patients were assessed at weekly intervals during the first month and then every 6 months by breast surgeons, plastic surgeons, and oncologists.

Major complications (requiring surgical revision), loco-regional recurrences (defined as local recurrence if involving the ipsilateral skin flap, chest wall, or NAC; or as regional recurrence if involving ipsilateral axillary, internal mammary, or supraclavicular nodes), cosmetic outcomes, quality of life, and economic costs were assessed in all patients.

An automated breast volume scanner (ABVS), a dedicated imaging system that can obtain full-field high-resolution views of skin flaps, was used to better evaluate possible local recurrence in the usually thicker skin flaps of patients with PP-IPBR [10].

The “QOL assessment PRO” is a questionnaire created through a multidisciplinary effort by all specialists working in the Breast Unit of Fondazione Policlinico Universitario Agostino Gemelli IRCCS. It was developed based on the experiences reported in the literature, and has been proficiently employed in our center for several years [12,13,14,15,16,17]. The questionnaire condenses in seven simple questions the essential patient-reported outcomes (PROs) involving pain, arm motility, aesthetic satisfaction, and general quality of life (QOL), and is therefore a practical tool that in our experience gives results more agreeable to patients than BREAST-Q, and increases their compliance to participate in the study [18]. The QOL assessment PRO was administered six months after surgery via a telephone call by a member of hospital staff, and consisted of five close-ended questions (requiring a yes/no answer) and two scoring questions (requiring a score between 0 and 5 as an answer) (Table 1).

Results were expressed as means with associated median and range. Statistical analysis was performed using SPSS (version 24.0 for Windows). A Fisher exact test was used for comparison of categorical variables. A *p*-value equal to or less than 0.05 was considered statistically significant. A cost analysis was performed according to a standardized method [19].

## 3. Results

Over the two-year study period from January 2018 to December 2019, 177 breast cancer patients with IPBR after NSM were included. SM-IPBR was performed in 95 cases, while PP-IPBR was performed in 82 cases. Patient characteristics are reported in Table 2. Ptosis degree, Rancati score, and intraoperative flap thickness assessment were decisive in determining the kind of reconstruction performed, and therefore differed significantly between the PP and SM group. The remaining aspects were similar in both populations. Adjuvant radiotherapy did not affect aesthetic and oncological outcomes.

The mean ages were 44 (28–73) and 47 (27–73) years respectively. After unilateral NSM, a simultaneous contralateral symmetrization procedure was deemed necessary and carried out in 44/44 (100%) patients of the SM group and in 2/55 (3.6%) patients of the PP group. The type of surgical treatment is summarized in Table 3.

### 3.1. Duration of Surgery and of Hospitalization

For patients undergoing unilateral NSM and IPBR, the mean total operative time was 319 min in the SM group and 247 min in the PP group; for patients undergoing bilateral NSM, it was 368 min and 306 min, respectively.

The longest surgery (510 min) was for a patient who underwent a transaxillary bilateral mastectomy with sentinel node biopsy, axillary dissection, and bilateral SM reconstruction. Operative times are summarized in Table 4. Length of hospitalization did not significantly differ between the two populations.

### 3.2. Perioperative and Oncological Outcomes

Median follow-up was similar: 20 (6–28) months in the SM group and 16 (5–28) months in the PP group. There was no significant difference in length of stay, overall major complication rates, and oncological outcomes between the two reconstructive cohorts.

Implant loss caused by infection was observed in one patient in the SM group (1.05%) and one patient in the PP group (1.2%). One patient in the PP group (1.2%) developed a full-thickness NAC necrosis that required secondary excision.

During follow-up, NAC recurrence occurred in one patient of the SM group (1.05%), while in the PP group, no local relapse was observed. Regional recurrences occurred in 2/95 (2.1%) patients in the SM group and in 1/82 patients (1.2%) in the PP cohort.

Regarding disease-free survival, one patient in the SM group with triple negative breast cancer developed brain metastases six months after surgery.

### 3.3. Cosmetic Outcomes and Health-Related Quality of Life

A total of 126/177 patients completed our survey assessing their postoperative quality of life (64.2% and 78%, respectively, for the SM and PP groups).

Statistically significant (*p* < 0.05) advantages in terms of cosmetic results, chronic pain, shoulder dysfunction, and skin sensibility were observed in the PP group.

A not statistically significant difference in favor of the PP group was shown for sports activity and sexual/relationship life (Table 5).

### 3.4. Economic Performance

Whenever a surgical procedure is performed, different resources (including personnel, equipment, facilities, time, and materials) are utilized. A cost analysis was performed according to a standardized method and direct cost comparison [19]. The analysis showed better economic performances in the PP group due to shorter operative times, less-frequent need of contralateral breast symmetrization, and less-frequent use of contralateral implants. The average savings with PP-IPBR were EUR 1503 for unilateral NSMs and EUR 1568 for bilateral procedures (Table 6).

## 4. Discussion

In our institution, we offer IPBR to all patients undergoing NSM. For many years, we have used only SM placement of the implants, but since 2016, we also started to perform PP-IPBR in selected cases, initially with ADM coverage and only recently without the use of matrices [2,6,20].

PP placement of the prosthesis into the space of the excised mammary gland allows a more natural breast appearance with a more harmonious breast slope and ptosis [21,22,23]. It also allows, in most cases of unilateral NSM, the avoidance of symmetrization procedures on the contralateral breast [24,25]. In our experience, a symmetrization procedure was performed for 44/44 (100%) patients in the SM group, compared to only 2/55 (3.6%) cases in the PP group with polyurethane-covered implants.

Initially, when performing PP-IPBR, we used ADM coverage of the implant. ADMs are biologic scaffolds of human, bovine, or porcine origin that lack immunogenic epitopes and are therefore easily revascularized and integrated into host tissue without encapsulation or contracture [23,24,25,26].

The use of ADM, however, may be hampered by surgical and economic issues. Some authors reported higher medical costs, with a variable additional expense between USD 2100 and USD 3400, depending on the size of the dermal sheet utilized [17,27].

For these reasons, in January 2018 we started to perform PP-IPBR using a Polytech implant with a micropolyurethane-foam-coated shell surface (microthane) that does not require further ADM coverage [2,5]. The 1.4 mm micropolyurethane sponge coating is reabsorbed by the body and contributes to form an ideal capsule that protects the implant and reduces capsular contracture, resulting in softer and more natural-appearing breasts. Furthermore, the extremely adherent texture of this implant reduces the risks of rotation and displacement, and consequently the possible need for revision surgery [5].

Careful patient selection and surgical conduct are mandatory to perform PP-IPBR successfully. This technique should be considered only for patients in which adequate thickness and perfusion of skin flaps can be ensured during mastectomy [2,24,28].

To minimize the risk of learning-curve-related complications and technical problems, we considered exclusion criteria of BMI > 30kg/m^2^, oversized breasts, ptosis of grade >2, obese patients, heavy smokers, and previous radiation therapy [24,29].

Regarding the surgical conduct, lateral–radial incisions or axillary or inframammary crease incisions are preferable in order to better preserve vascular integrity of the NAC [20,29,30]; skin flaps of adequate thickness should be separated from the mammary gland using blunt dissection and preserving medial perforators, and real-time skin-perfusion testing with a fluorescence imaging system should be performed intraoperatively to assess skin-flap viability with immediate resection of potential ischemic tissues. Choice of implant size and shape should be based on evaluation of the breast and chest-wall conformation and accurate weight of the surgical specimen (in this regard, we recommend using fill volumes similar to those of the removed gland).

With proper patient and implant selection and careful surgical conduct, PP-IPBR can be performed with results similar to SM-IPBR in terms of postoperative complication rates and oncologic safety [1,27,30,31]. In our series, there were no statistically significant differences in terms of implant failure and local, regional, or systemic recurrence between the two groups. We observed only two cases of major complications that led to implant loss: one case of infection in the PP group, and one in the SM group. One patient in the PP group developed NAC necrosis. We classified this complication as minor because it required no surgical revision and was treated successfully in outpatient regime, as the necrosis involved only a small portion of the NAC and was not full thickness.

Regarding patient quality of life, we observed statistically significant improvements in aesthetic results, chronic pain, shoulder dysfunction, and skin sensibility (*p* < 0.05) in the PP group, and a trend of better outcomes (even if statistically not significant) regarding sports activity and sexual/relationship life in this group.

These better results are probably explained by the avoidance of chest-wall musculature manipulation in PP-IPBR [1,2,5].

PP-IPBR significantly reduces operative time as there is no need for submuscular pocket creation, and, in most cases, for contralateral breast symmetrization. When using microthane-coated implants, operative time is further reduced by the avoidance of ADM coverage [2,5,27].

In our series, this shorter operative time, coupled with the reduced need for contralateral implants, generated an average saving of EUR 1500 for unilateral procedures; this saving significantly increased when using a Polytech implant, as the costs of ADM coverage are also avoided (the cost of a 30 × 20 cm sheet of ADM in our hospital is EUR 4056). Furthermore, because PP reconstruction averts the issues related to pectoralis major muscle manipulation, it also minimizes postoperative costs of painkillers and postoperative physiotherapy, with additional benefit for the healthcare system [32,33,34,35].

## 5. Conclusions

Our study presents several limitations, as it is a retrospective unicentric analysis with a relatively limited duration of follow-up, and may include a small selection bias, as PP-IPBR without ADM has been adopted in our institution only recently, and therefore grants less expertise and more potential for technical mistakes than SM-IPBR. However, this work provides encouraging preliminary data on the safety and efficacy of PP positioning of microthane-coated implants without ADM in patients undergoing NSM with IPBR.

PP-IPBR can represent a valid alternative to traditional IPBR, improving outcomes and patient quality of life; it is easier to perform, reduces operative time, and minimizes complications related to manipulation of the pectoralis major muscle, while also contributing to the containment of costs.

Careful patient selection, adequate surgical experience, and repetitive practice of specific tasks are mandatory to optimize the outcomes and reduce the risk of minor and major complications. Further prospective trials with a larger number of patients and a longer follow-up are necessary to draw more validated conclusions.

## Figures and Tables

**Table 1:** QOL assessment PRO survey.

Smart QoL Assessment
Quality of life
What score would you give to your pain, from 0 (no pain) to 5 (very intense)?
Is arm motility impaired after surgery? (YES/NO)
Did you do sports before surgery? (YES/NO) Have you practiced sports since surgery? (YES/NO)
Was the sensitivity of the skin and the areola-nipple complex maintained after surgery? (YES/NO)
Satisfaction
How would you evaluate, from 1 (poor) to 5 (excellent), the aesthetic result of your operation?
Psychological and relational field
Did the operation compromise your womanhood, sexuality, or relationship life? (YES/NO)

Abbreviations: QOL = quality of life; PRO = patient-reported outcomes.

**Table 2:** Patient characteristics.

Characteristics	PP-IPBR	SM-IPBR	*p*
Patients, *n* (%)	82 (46.3%)	95 (53.6%)	
Age (years)	47 (27–73)	44 (28–73)	0.113
FUP (months)	15.9 (5–28)	20 (6–28)	0.254
Radiotherapy adjuvant, *n* (%)	23/82 (28.0%)	22/95 (23.2%)	0.355
Body Mass Index (BMI)	23.95 (17.5–29.4)	24.77 (18.2–28.9)	0.135
Neoadjuvant chemotherapy, *n* (%)	35/82 (42.7%)	38/95 (40.0%)	0.938
BRCA 1/2 mutation, *n* (%)	13/82 (15.8%)	12/95 (12.6%)	0.539
Ptosis degree			**<0.001**
⮚ 0	19 (23.2%)	41 (43.2%)	
⮚ 1	31 (37.8%)	48 (50.5%)	
⮚ 2	32 (39%)	6 (6.3%)	
Rancati score			**<0.001**
⮚ 1	0	36 (37.9%)	
⮚ 2	44 (53.7%)	38 (40%)	
⮚ 3	38 (46.3%)	21 (22.1%)	
Intraoperative flap thickness assessment (Indocyanine green visualization < 60 s)	82 (100%)	12 (12.6%)	**<0.001**

Abbreviations: FUP = follow-up; BRCA = breast cancer gene. Statistically significant *p* values (<0.05) are marked in bold.

**Table 3:** Type of surgical treatment.

Surgical Procedures	PP-IPBR	SM-IPBR	*p*
Bilateral NSM with IPBR, *n* (%)	27/82 (32.9%)	51/95 (53.7%)	**0.006**
Unilateral NSM with IPBR, *n* (%)	55/82 (67.1%)	44/95 (46.3%)	**0.008**
Contralateral implant-based symmetrisation mammoplasty after unilateral NSM with IPBR, *n* (%)	2/55 (3.6%)	44/44 (100%)	**<0.001**

Abbreviations: NSM = nipple-sparing mastectomy; IPBR = immediate prosthetic breast reconstruction. Statistically significant *p* values (<0.05) are marked in bold.

**Table 4:** Operative time (minutes).

	PP-IPBR	SM-IPBR	*p*
Bilateral NSM + IPBR	306 (202–381)	368 (276–478)	**0.041**
Unilateral NSM + IPBR + contralateral symmetrization	247 (182–305)	319 (254–393)	**<0.001**

Statistically significant *p* values (<0.05) are marked in bold.

**Table 5:** QOL assessment PRO survey replies.

	N° Total Patients	PP-IPBR	SM-IPBR	*p*
**Patients who completed our survey**	126/177 (71.2%)	64/82 (78%)	62/95 (64.2%)	
**Aesthetic satisfaction**				**<0.001**
VOTE 1 (poor)	5	0 (0%)	5 (8.1%)	
VOTE 2 (insufficient)	9	1 (1.6%)	8 (12.9%)	
VOTE 3 (satisfactory)	33	4 (6.3%)	29 (46.8%)	
VOTE 4 (good)	30	17 (26.6%)	13 (21.0%)	
VOTE 5 (excellent)	49	42 (65.6%)	7 (11.3%)	
**Skin sensibility**				**0.025**
YES	49	31 (48.4%)	18 (29.0%)	
NO	77	33 (51.6%)	44 (71.0%)	
**Compromised relationship life**				0.208
YES	42	18 (28,1%)	24 (38,7%)	
NO	84	46 (71,9%)	38 (61,3%)	
**Sports before surgery**				0.472
YES	65	31 (48.4%)	34 (54.8%)	
NO	61	33 (51.6%)	28 (45.2%)	
**Sports after surgery**				0.881
YES	52	26 (40.6%)	26 (41.9%)	
NO	74	38 (59.4%)	36 (58.1%)	
**Chronic pain in pectoral region**				**<0.001**
0 (no pain)	40	32 (50.0%)	8 (12.9%)	
1 (very mild)	24	19 (29.7%)	5 (8.1%)	
2 (mild)	16	8 (12.5%)	8 (12.9%)	
3 (tolerable)	31	4 (6.3%)	27 (43.5%)	
4 (distressing)	12	1 (1.6%)	11 (17.7%)	
5 (very intense)	3	0 (0%)	3 (4.8%)	
**Impaired arm motility**				**<0.001**
YES	28	3 (4.7%)	25 (40.3%)	
NO	98	61 (95.3%)	37 (59.7%)	

Statistically significant *p* values (<0.05) are marked in bold.

**Table 6:** Economic analysis.

Costs			
	PP-IPBR	SM-IPBR	*p*
OR cost for unilateral NSMs (EUR 6.5/min)	EUR 1605.5	EUR 2073.5	**<0.001**
OR cost for bilateral NSMs (EUR 6.5/min)	EUR 1989	EUR 2392	**<0.001**
Implant (EUR 1100/implant)	EUR 1100	EUR 2200	**<0.001**
**Savings**			
**PP-IPBR without ADM vs. SM-IPBR**	Unilateral NSM	Bilateral NSM	
	EUR 1503	EUR 1568	0.543

Abbreviations: PP-IPBR = prepectoral immediate prosthetic breast reconstruction; SM-IPBR = submuscular immediate prosthetic breast reconstruction; OR = operating room; NSM = nipple-sparing mastectomy; ADM = acellular dermal matrix. Statistically significant *p* values (<0.05) are marked in bold.

## Data Availability

The data presented in this study are available on request from the corresponding author.
